# The Microbial Community of the Cystic Fibrosis Airway Is Disrupted in Early Life

**DOI:** 10.1371/journal.pone.0109798

**Published:** 2014-12-19

**Authors:** Julie Renwick, Paul McNally, Bettina John, Todd DeSantis, Barry Linnane, Philip Murphy, on behalf of SHIELD CF

**Affiliations:** 1 Department of Clinical Microbiology, Trinity College, Dublin, Ireland; 2 The National Children's Research Centre, Our Lady's Children's Hospital, Dublin, Ireland; 3 The National Children's Hospital, Tallaght hospital, Dublin, Ireland; 4 Second Genome, South San Francisco, California, United States of America; 5 Hospital Limerick, Limerick, Ireland; 6 Centre for Interventions in Infection, Inflammation & Immunity (4i), Graduate-Entry Medical School, University of Limerick, Limerick, Ireland; Texas Tech University Health Sciences Center, United States of America

## Abstract

**Background:**

Molecular techniques have uncovered vast numbers of organisms in the cystic fibrosis (CF) airways, the clinical significance of which is yet to be determined. The aim of this study was to describe and compare the microbial communities of the lower airway of clinically stable children with CF and children without CF.

**Methods:**

Bronchoalveolar lavage (BAL) fluid and paired oropharyngeal swabs from clinically stable children with CF (n = 13) and BAL from children without CF (n = 9) were collected. DNA was isolated, the 16S rRNA regions amplified, fragmented, biotinylated and hybridised to a 16S rRNA microarray. Patient medical and demographic information was recorded and standard microbiological culture was performed.

**Results:**

A diverse bacterial community was detected in the lower airways of children with CF and children without CF. The airway microbiome of clinically stable children with CF and children without CF were significantly different as measured by Shannon's Diversity Indices (p = 0.001; t test) and Principle coordinate analysis (p = 0.01; Adonis test). Overall the CF airway microbial community was more variable and had a less even distribution than the microbial community in the airways of children without CF. We highlighted several bacteria of interest, particularly *Prevotella veroralis*, CW040 and a *Corynebacterium*, which were of significantly differential abundance between the CF and non-CF lower airways. Both *Pseudomonas aeruginosa* and *Streptococcus pneumoniae* culture abundance were found to be associated with CF airway microbial community structure. The CF upper and lower airways were found to have a broadly similar microbial milieu.

**Conclusion:**

The microbial communities in the lower airways of stable children with CF and children without CF show significant differences in overall diversity. These discrepancies indicate a disruption of the airway microflora occurring early in life in children with CF.

## Introduction

The long held concept that the lower airway is a sterile environment has been challenged recently [1], [2]. New molecular techniques are not only demonstrating the presence of organisms where previously there were considered to be none, but are finding a large variety of taxa present at any one time in the “normal” lung [1]–[3]. In the respiratory tract, as in other organ systems, there appears to be a more diverse array of organisms in health than in disease [1], [4], [5]. These challenging discoveries are forcing a paradigm shift in our approach to understanding the concepts of colonisation and infection in the lung. We are now faced not simply with an infecting pathogen, but instead with a diverse community of organisms where disease may be defined by the makeup and relative abundance of the members of the microbial community [6].

The evolution of the airway microbiome in cystic fibrosis (CF) and its relationship to lung disease progression is still poorly understood. Traditionally, CF antimicrobial therapy has been directed against a small number of common pathogens detected by standard culture. Recent evidence has revealed hundreds of bacteria to be present in the CF airways [7], [8], raising questions about the relevance of current diagnostic methods. A considerable variation in species' drug susceptibility [9]–[11] and virulence [12] is likely to exist in these microbial communities and directing anti-microbial therapy towards one or two pathogens has unknown consequences on the remaining microbial community. Cross-sectional and longitudinal studies examining the impact of antibiotics on the CF airway microbiome have reported an association between antibiotic exposure and a transient decrease in microbiome diversity [6], [13]. However microbial community structures in the CF airway have been reported to return to pre-exacerbation compositions within four weeks of antibiotic treatment and furthermore airway microbial community structures within individual CF patients have been shown to be resilient over time despite antibiotic treatment [14]. The types of species present in the airway microbial community and the relative abundance of these species may be as important as the presence or absence of common pathogens in the CF airway.

Our study employed a 16S rRNA microarray to describe and compare the microbial community in the lower airways of children with CF and without CF (controls) in an attempt to understand more clearly the abnormal microbiological milieu in the CF airway. An increase in our understanding of this complex area may help us to discover more relevant diagnostic tests and develop more appropriate antimicrobial regimes for children with CF.

## Materials and Methods

### Ethics Statement

Ethical approval for this study was granted by Our Lady's Children's Hospital Crumlin research ethics committee (reference number GEN/210/11) and informed consent was obtained from the parents or guardians of all participating subjects.

### Patient Group

Infants and young children were recruited through the Study of Host Immunity and Early Lung Disease in CF (SHIELD CF), a longitudinal study established around a preschool bronchoalveolar (BAL) surveillance programme at our institutions. This involves performing bronchoscopy and BAL on clinically stable children up to the age of 6 years (or until the child is expectorating) as part of an annual review. Stability was defined as no change in respiratory symptoms from baseline or use of antibiotics for infective exacerbations within 3 weeks of the procedure. Patients in this study were recruited over a 13 month period between October 2010 and November 2011.

Children without CF undergoing bronchoscopy for clinical reasons, such as prior haemoptysis or stridor, were also recruited. These children were clinically well at the time of the procedure. The BAL samples from this control cohort were included for comparison if the differential cell count in BAL was normal.

### BAL and Oropharyngeal Swab Collection and Storage

Flexible fibreoptic bronchoscopy was performed via a laryngeal mask airway under general anaesthetic. To prevent upper airway contamination, suction was not performed until the tip of the bronchoscope was past the carina. BAL was performed by instilling 1 ml/kg sterile 0.9% saline (w/v) per aliquot and retrieved using low pressure suction. The BAL was performed twice in the right middle lobe and twice in the lingula. All four samples were then pooled. BAL was immediately sealed in a sterile container, put on ice and transported to the laboratory. An aliquot of BAL fluid was sent directly for standard microbiological testing. The remaining BAL fluid was split and one part treated with two volumes of RNALater (Qiagen). The remainder was processed as follows: Total cell counts were performed using trypan blue exclusion and differential cell counts were carried out manually on cytospin preparations of BAL by light microscopy. BAL was subsequently centrifuged at 1,057×g and the supernatant aliquoted and frozen at −80°C for subsequent experiments. Oropharyngeal swabs (SWB) were collected using a sterile double-tipped swab (BBL CultureSwab, Becton, Dickinson) by a CF nurse specialist experienced in the procedure. One tip was sent for standard culture and the other tip was suspended in 2 ml RNALater. Samples were then stored at −80°C.

### Standard Microbiological Culturing

Health Protection Agency protocols were adhered to for all microbiological testing. Selective media for the detection and enumeration of *Staphylococcus aureus* (Colistin DNase Mannitol agar), Methicillin resistant *S. aureus* (salt enrichment broth), *Burkholderia cepacia* (*Burkholderia cepacia* selective agar), *Haemophilus influenzae* (Bacitracin chocolate agar) and *Pseudomonas aeruginosa* (Cetrimide agar) were employed. Additionally, chromogenic selective agar, blood agar, chocolate agar and MacConkey agar were used to identify additional species such as *Streptococcus pneumoniae*, *Candida* species and *Moraxella catarrhalis*. To provide semi-quantitative information and to provide a standard system for comparing culture data to microbiome diversity, growth was scored as absent (0), 1 to 100 CFU/ml (1), 100 to 10,000 CFU/ml (2) or >10,000 CFU/ml (3).

### Isolation and Quantification of DNA from BAL and SWB

Frozen BAL samples were thawed on ice and centrifuged for 10 min at 10,000×g. SWB samples were thawed, mixed by inverting and centrifuged for 10 min at 10,000×g. The pelleted material was then resuspended in 600 µl of buffer RLT (Qiagen DNA/RNA AllPrep Mini kit) and bead beating performed (0.3 g of 180 µm acid washed glass beads; Sigma Aldrich) for 30 sec at 5.5 m sec^−1^. Supernatant was transferred to a Qiagen DNA spin column and DNA extracted using the Qiagen DNA/RNA AllPrep Mini kit. DNA quantity was determined using the Quant-iT PicoGreen dsDNA assay kit (Invitrogen) following the manufacturer's instructions. A negative control was included where the entire DNA extraction process was performed with 1 ml of 0.9% saline (w/v) as the start material. No DNA was isolated from the 0.9% saline (w/v) however 16S rRNA amplification was still performed on this negative control.

### Amplification of the 16S rRNA region

The bacterial 16S rRNA region was amplified using universal 16S rRNA primers 27F (5′-AGAGTTTGATCCTGGCTCAG-3′) and 1492R (5′-GGTTACCTTGTTACGACTT-3′) as previously described [7]. Each sample and the negative control was amplified in four replicate reactions over a range of annealing temperatures. Thermal cycling parameters were as follows; 95°C for 3 min for 1 cycle followed by 30 cycles of 95°C for 30 sec, 46–56°C for 25 sec, 72°C for 2 min and a final extension step of 72°C for 10 min. For each sample, amplicons were pooled, purified with the QIAquick PCR purification kit (Qiagen, Valencia, CA), concentrated using the solid-phase reversible immobilization method and quantified by electrophoresis (Agilent 2100 Bioanalyzer). The 0.9% saline (w/v) negative control yielded no 16S rRNA PCR amplicon. Phylochip Control Mix was added to each amplified product allowing quantitative interpretation of the relative abundance of taxa.

### Phylochip Microarray

For the analysis of bacterial communities the G3 Phylochip Array (Second Genome, San Bruno, CA) was employed. A full description of the Phylochip design is provided in the supplementary methods of Hazen *et al*. [15] and are briefly outlined here in S1 File: Supplementary Methods. Ribosomal PCR products were DNAase fragmented, biotin labelled, and hybridized to the G3 Phylochip Array, then washed, stained, and scanned using a GeneArray scanner (Affymetrix) as previously described [15].

### Microarray Data Processing

The summary intensity for each feature on each array was calculated by ranking the central 9 pixels of individual features by intensity and the 75^th^ percentile was used. Probe intensities were background-subtracted within each of the 25 separate physical sectors and scaled to the Phylochip Control Mix. Array fluorescence intensities were collected as integer values ranging from 0 to 65,536 (2^16^). Presence/Absence scoring was conducted according to the positive fraction approach. Noise (N) for each array was calculated according to DeSantis 2007 [16]. For each taxa represented on the Phylochip a perfectly matched (PM) probe and mismatched (MM) probe were incorporated. A PM probe is complementary to the target sequence, whereas the MM probe is identical in all of the 25 nucleotide positions except the thirteenth base. Fluorescence intensity from PM and MM probes were compared and considered “positive” when PM-MM > = 50*N and PM/MM > = 1.5. PM fluorescence intensities from 73,959 probes observed as positive in at least 3 experiments were exported from all experiments then ranked within each sample and used as input to empirical probe-set discovery by cluster analysis. Probes were clustered into probe-sets or empirical Operation Taxonomic Unit (eOTU) based on both correlations in fluorescence intensities across all biological samples and taxonomic relatedness. Where multiple clustering solutions were available, higher correlation coefficients were favoured over lower coefficients, taxonomic relatedness at the species level was favoured over higher ranks, and sets composed of more probes were favoured over less. All probe sets contained > = 7 probes, with average pair-wise correlation coefficients > = 0.85. The eOTU tracked by a probe set was taxonomically annotated from the combination of the 9-mers contained in all probes of the set. The mean ranked fluorescence intensity for each eOTU and each sample was calculated. These values are referred to as the hybridization scores (HybScore) used in abundance-based analysis. eOTUs were considered present in a sample if > = 80% of their probes were positive for that sample. A more detailed description of the microarray data processing and data reduction is in S1 File: Supplementary Methods.

### Microarray Data Analysis

Microbial communities are profiled and compared based on diversity. Microbial communities can be described based on the number of taxa present (richness), the quantity of the bacterial taxa present (abundance) and the relative abundance of the taxa present (evenness). After the taxa are identified for inclusion in the analysis, the values used for each taxa-sample intersection are populated in two distinct ways; the abundance metrics and the incidence metrics. Taxa are then filtered to those present in at least one of the samples or to taxa significantly increased in their abundance in one category compared to the alternate categories. For the latter filter, the parametric Welch test was employed to calculate p-values. Additionally, q-values were calculated using the Benjamini-Hochberg procedure to correct p-values, controlling for false discovery rates. Multiple testing was also accounted for using permutation tests.

To compare sample-to-sample distances, profiles were inter-compared in a pair-wise fashion to determine a dissimilarity score which was stored in a distance dissimilarity matrix. The distance functions were chosen to allow similar biological samples to produce only small dissimilarity scores. The UniFrac distance metric [17], which utilizes the phylogenetic distance between eOTUs, was used to determine the dissimilarity between communities. Additionally, Weighted Unifrac was employed to consider eOTU abundance. Principal Coordinate Analysis (PCoA) biplots were constructed using UniFrac and weighted UniFrac dissimilarity matrices to visualize complex relationships between samples. The Adonis test, a Monte Carlo permutation test, was performed on distance matrices to compare the between category differences of randomly reassigned sample categories to the true categories. M Diversity indices such as the Shannon's Diversity Index (SDI) provide a mathematical measure for describing the diversity of a community taking in to consideration species richness and differences in abundances for each species across samples [18]. The proportion of a species relative to the total number of species is computed as the ratio of the species HybScore to the sum of HybScores. In molecular profiling, the relative value of the SDI is compared between sample groups to infer differences in underlying microbial community. Microsoft Excel was used to calculate the SDI of the microbial community present in each sample.

To construct the circular tree, a Welch test was performed across the groups of samples using the abundance metrics. Those taxa significantly below the threshold (p<0.05) were grouped into families and the one taxon from each family (plus one if a family contained eOTUs that significantly increased and decreased in abundance between categories) with the greatest difference between the two group means was selected for inclusion in the circular tree. 59 taxa from 51 families had significantly increased or decreased abundance in one of the comparison groups. The tree, taxonomy labels and abundance data were rendered in iTOL. The Adonis test was then used to find significant differences among discrete categorical or continuous variables (age, sex, *P. aeruginosa, H. influenzae, S. pneumoniae, M. catarrhalis, Candida*). The PhyCA-Stats analysis software package was used for multivariate analysis and R software version 3.0.1 was used to carry out all other analysis.

The raw hybridisation data, processed hybridisation data (abundance metrics and binary metrics), eOTU annotations and metadata associated with this study were deposited in Greengenes database and can be accessed at the following link http://greengenes.secondgenome.com/downloads/phylochip_datasets.

## Results

Thirteen clinically stable children with CF and nine control children were recruited. Baseline clinical characteristics of the study group are presented in Table 1. The mean age of the CF patient cohort was 3.95 years (ranging 1.1 to 8.1 years) and the mean age of the non-CF patient cohort was 3.78 years (ranging 0.9 to 9.75 years). All CF patients had no change in respiratory symptoms from baseline and were not receiving antibiotics for up to three weeks prior to bronchoscopy. All non-CF control patients were clinically well at the time of bronchoscopy and had baseline normal differential cell count in BALs (as compared to historical true controls; data not shown).

**Table 1:** Baseline demographic and clinical characteristics of all patients included in the study.

Patient number	CF/Control	Sex (M/F)	Age (years)	CFTR mutation	Culture data (0 to 3)						Medications/supplements at time of bronchoscopy
					PA	SP	MC	SA	HI	NF	
1	CF	M	2.18	ΔF508/ΔF508	0	0	0	0	0	3	Creon, vitamins, Omeprazole, HS
2	CF	F	5.3	ΔF508/ΔF508	0	0	0	1	3	1	Creon, vitamins, HS
3	CF	M	3.75	ΔF508/ΔF508	0	0	0	0	0	1	Creon, vitamins, HS
4	CF	F	1.1	ΔF508/ΔF508	2	0	0	0	0	1	Creon, vitamins
5	CF	F	5.6	ΔF508/ΔF508	0	0	0	0	0	2	Creon, vitamins, HS
6	CF	M	3.25	ΔF508/ΔF508	0	0	0	0	2	1	Creon, vitamins, HS
7	CF	M	4.7	ΔF508/ΔF508	0	1	0	1	1	1	Creon, vitamins, HS
8	CF	M	3	ΔF508/ΔF508	0	0	1	0	1	1	Creon, vitamins, HS, DNAse
9	CF	M	3.25	ΔF508/ΔF508	0	0	0	0	3	1	Creon, vitamins
10	CF	M	2.85	ΔF508/ΔF508	3	2	0	0	0	1	Creon, vitamins
11	CF	M	5.3	ΔF508/2184DelA	0	0	0	0	3	1	Creon, vitamins, HS
12	CF	M	8.1	ΔF508/ΔF508	0	0	0	1	1	2	Creon, vitamins, HS
13	CF	F	3	ΔF508/ΔF508	0	0	0	0	0	3	Creon, vitamins, HS
14	Control	F	1.7	N/A	0	0	2	1	2	0	No medication
15	Control	F	2	N/A	0	0	0	0	0	0	No medication
16	Control	F	2	N/A	0	3	0	0	3	1	ICS
17	Control	F	8	N/A	0	0	0	0	1	1	No medication
18	Control	F	1.92	N/A	0	0	0	0	3	1	No medication
19	Control	M	4	N/A	0	0	0	0	3	1	ICS
20	Control	M	0.9	N/A	0	0	0	0	1	1	No medication
21	Control	M	9.75	N/A	0	0	0	0	0	1	No medication
22	Control	F	3.75	N/A	0	0	0	0	3	1	ICS

*PA  =  Pseudomonas aeruginosa, SP  =  Streptococcus pneumoniae, MC  =  Moraxella Catarrhalis, SA  =  Staphylococcus aureus, HI  =  Haemophilus influenzae, NF  =  Normal Flora, ICS  =  Inhaled corticosteroids, HS  =  Hypertonic Saline.*

*0 = 0 CFU/ml, 1 = 1 to 100 CFU/ml, 2 = 100 to 10,000 CFU/ml and 3 = >10,000 CFU/ml.*

### The control and CF lower airway microbial communities differ significantly while the upper and lower CF airway communities are similar

A total of 73,959 probe pairs were positive over our patient population. From this 1,269 were detected in at least one sample using the microarray. An average of 167 species (ranging 145 to 202) were identified from the control lower airway, 133 species (ranging 67 to 163) from the CF lower airway and 130 species (ranging 71 to 167) from the CF upper airway. In descending order, the phyla accounting for the majority of the fluorescence intensity were Proteobacteria, Firmicutes, Bacteroidetes, an unclassified bacterial Phylum and Tenericutes. The genera accounting for the majority of the fluorescence intensity were an unclassified *Lachnospiraceae* genus, an unclassified *Rikenellaceaell* genus, *Pseudomonas*, *Streptococcus* and *Prevotella*.

**Figure 1:**
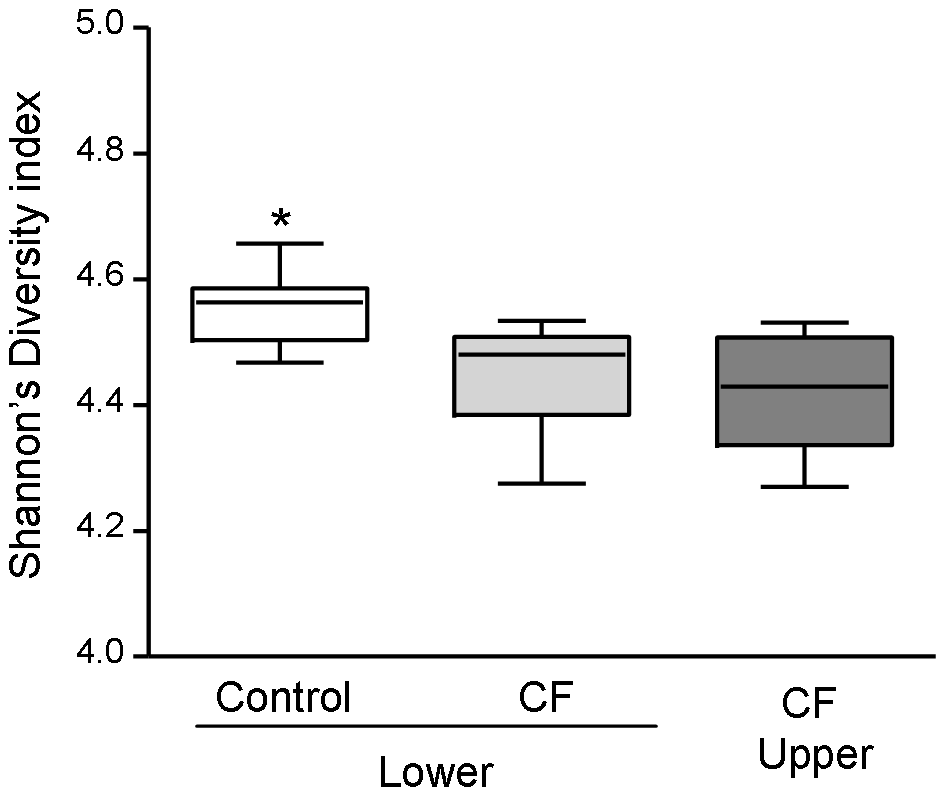
Richness of the microbial communities present in the control lower airways and the CF upper and lower airways. Box and Whiskers plot of Shannon's diversity indices for microbial communities present in the control lower airway, CF lower airway and the CF upper airway. * represents statistical significance (p = 0.001; two-tailed t test).

SDI's were calculated for each sample and used to compare overall diversity of the three study groups. The control lower airway bacterial community was significantly more diverse than that of the CF lower airway (p = 0.001; heteroscadastic, two-tailed t-test). The CF upper and lower airway microbiome did not differ significantly in their overall diversity (p = 0.534; heteroscadastic, two-tailed t-test) (Fig. 1).

**Figure 2:**
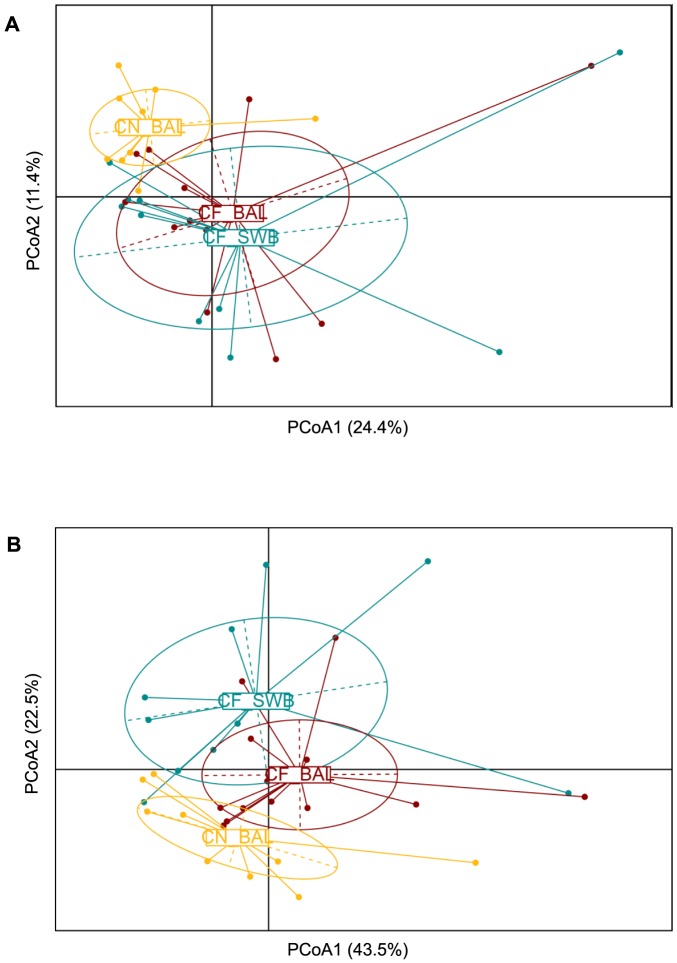
Bacterial community structures in the control lower airways and the CF upper and lower airways. Principle coordinate analysis (PCoA) plots of A) UniFrac distances and B) weighted UniFrac distances of all airway microbial communities were created in R. Each community from each sample is represented as a filled circle and coloured by sample type and/or patient cohort; CF lower (CF BAL; red), CF upper (CF SWB; blue) and control lower (CN BAL; yellow) airway samples. The x-axis and y-axis represent 2-dimensions of percentage variation explained by the PCoA. Ellipses were included for visualisation purposes.

Principal Coordinate Analysis (PCoA) is a method of two-dimensional ordination plotting that is used to visualize complex relationships between samples. PCoA uses dissimilarity values generated by calculating distance measures to position the points relative to each other on a bi-plot. Separate distance matrices based on taxa presence/absence and taxa abundance were constructed for each patient cohort and UniFrac and weighted UniFrac distance measures, respectively, were used to plot the dissimilarity between microbial communities on PCoA bi-plots. The Adonis test was performed on distance matrices to compare the between category differences of randomly reassigned sample categories to the true categories. The airway bacterial communities from our three categories differ significantly based on taxa present (p = 0.015) (Fig. 2A) and on taxa abundance (p = 0.020) (Fig. 2B). Additional Adonis tests were performed to compare the individual groups. The control lower and CF lower airway samples grouped significantly based on taxa present (p = 0.01), however the CF lower and CF upper airway samples did not group significantly based on taxa present (p = 0.292) or taxa abundance (p = 0.116). These results demonstrate that the CF airway microbial community structure is disrupted from the control state. These results also reveal that the structure of the microbial community in the CF upper and lower airways are broadly similar, suggesting that both specimens may be valid representatives of the CF airway microbial community.

### Taxa of significance between the control and CF lower airway microbial communities

**Figure 3:**
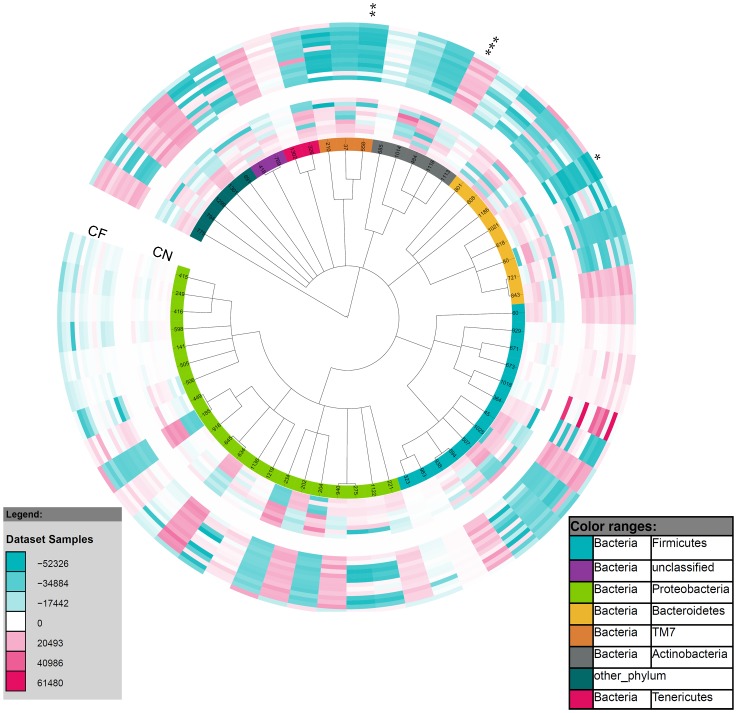
Taxa of differential abundance between the control and CF lower airway. Circular Phylogenetic Tree was rendered in iToL and illustrates abundance changes between eOTUs present in the control lower (inner ring) and CF lower (outer ring) airways. The colour saturation indicates the degree of difference from the mean control lower value for the eOTU; where dark blue indicates a HybScore difference of −52326, white  = 0 (no change from mean control lower), dark red  = +61480. Two eOTUs remained of significantly differential abundance following Benjamini-Hochberg correction; *Prevotella veroralis* (*) and a CW040 (**). Following a permutation test, 62 taxa were found to be of significantly differential abundance, *Corynebacterium* (***) is highlighted. A detailed list of the 59 taxa of significantly differential abundance between categories is provided in the supplementary data (S2 File).

There were 237 taxa found to be present in significantly different abundance between the control and CF lower airways using the Welch test (p<0.05) (S2 File). Fifty-nine eOTUs within 50 families were significantly different (p<0.05) in one of the comparison groups (Fig. 3). When the Benjamini-Hochberg procedure was performed to adjust for multiple testing, only 2 eOTUs remained significant (q<0.05); *Prevotella veroralis* within the Bacteroidetes phylum and CW040 within the TM7 phylum. Both of these eOTUs were significantly less abundant in the CF airway. The Benjamini-Hochberg procedure is a harsh method for adjusting for multiple testing therefore a permutation test was performed to determine how many of the 237 eOTUs would pass the Welch test at p<0.05 by randomizing the abundance table 100 times (S3 File). Following this randomized permutation test the median randomization found 41 eOTUs as significantly different, far fewer than the 237 eOTUs that were found in the real data. The “best” result from the randomized data was 175 eOTUs. Therefore the 62 eOTUs with the lowest p-values were considered to be of significantly differential abundance between the control and CF lower airway (S2 File). From these eOTUs, a *Corynebacterium* was found to be more abundant in the CF airway than in the control airway following the permutation test (Fig. 3). Other genera found to be of significantly differential abundance between the control and CF airway were *Prevotella, Bacteroides, Pseudomonas, Streptobacillus, Actinomyces, Selenomonas, Blautia* and *Leptotrichia*.

The individual eOTUs were investigated for their specific alliance with one or the other category (CF and control). In total, 58 eOTUs were found to be present in >30% of one category while being completely absent from the other category (S4 File). A *Streptococcus* sp, an unclassified Bacteria and a *Corynebacterium* sp were present in >46% of CF lower airway samples while completely absent from the control lower airway samples (Table 2). A *Pseudomonas* sp, three unclassified Lachnospiraceae and *Peptostreptococcus anaerobius* were present in >44% of the control airway samples while absent from the CF airway samples. While 4 eOTUs, including *Prevotella veroralis*, were found to be present in all but three control lower airway samples but absent in all CF lower airway samples (Table 2). The bacteria highlighted in this section may describe the major niches of disruption that cause an imbalance of the CF airway microbial community.

**Table 2:** eOTUs present in one category and absent from the other.

Phylum	Family	Genus	Species	Present in control (%)	Present in CF (%)
Proteobacteria	Procobacterieceae	Procobacter	Unclassified	0	38.5
Tenericutes	Acholeplasmataceae	Candidatus	Candidatus phytoplasma	0	38.5
Bacteroidetes	Unclassified	Unclassified	Unclassified	0	38.5
Firmicutes	Streptococcaceae	Streptococcus	Unclassified	0	46
Unclassified	Unclassified	Unclassified	Unclassified	0	46
Actinobacteria	Corynebactericeae	Corynebacterium	Unclassified	0	46
Firmicutes	Lachnospiriceae	Unclassified	Unclassified	55.6	0
Bacteriodetes	Rickenellaceaell	Unclassified	Unclassified	55.6	0
Proteobacteria	Pseudomonaceae	Pseudomonas	Unclassified	55.6	0
Unclassified	Unclassified	Unclassified	Unclassified	55.6	0
Proteobacteria	Comamonadaceae	Diaphorobacter	Unclassified	55.6	0
Firmicutes	Veillonellaceae	Unclassified	Unclassified	55.6	0
Fusobacteria	Fusobactericeae	Leptotrichia	Unclassified	55.6	0
Firmicutes	Veillonellaceae	Selenomonas	Unclassified	55.6	0
TM7	F16	Unclassified	Unclassified	55.6	0
Actinobacteria	Actinomycetaceae	Actinomyces	Unclassified	66.7	0
TM7	Unclassified	Unclassified	Unclassified	66.7	0
Proteobacteria	Unclassified	Unclassified	Unclassified	66.7	0
Bacteroidetes	Prevotellaceae	Prevotella	Prevotella veroralis	77.8	0

### Bioburden of *Pseudomonas aeruginosa* and *Streptococcus pneumoniae*, as determined by culture, was associated with bacterial community diversity

*P. aeruginosa* and *S. pneumoniae* presence and burden, as quantified by culture, were the only discrete variables associated with overall bacterial community diversity in the CF lower airways (Table 3). An Adonis test was performed using the UniFrac distance matrix and weighted UniFrac distance matrix to determine if samples with dissimilar *S. pneumoniae* and *P. aeruginosa* culture scores were dissimilar in their whole microbiome profile. CF patients with dissimilar *P. aeruginosa* culture scores had significantly dissimilar microbial community structure (p = 0.011 and p = 0.009 for community evenness and richness respectively). Likewise, CF patients with dissimilar *S. pneumoniae* culture scores had dissimilar microbial community structure (p = 0.035 for community richness). Age was not associated with community diversity in the CF airway which has been noted previously [6] however our patient cohort was of a young age only. From this study it was also noted that the *Pseudomonas* and *Haemophilus* genera had an inverse correlation (r = −0.703) across all samples in the study as measured by Pearson's correlation analysis on the sum of HybScores for all eOTUs belonging to these two genera. This suggests that these two genera may be competitors in the airway microbiome.

**Table 3:** Adonis scores for correlations between CF community diversity and discrete variables.

Variable	Community evenness	Community richness
Sex	0.950	0.920
Age	0.160	0.233
CFTR mutation	N/A	N/A
*P. aeruginosa*	0.011*	0.009*
*S. pneumoniae*	0.062	0.035*
*M. catarrhalis*	0.843	0.825
*S. aureus*	0.583	0.606
*H. influenzae*	0.307	0.465

*The CFTR mutation category was Not Applicable (N/A) as all but one patient was F508del homozygous.*

* Discrete variables that passed the Adonis test (p<0.05).

## Discussion

Here we describe the microbial milieu of the lower airways of clinically stable children with CF and compare it to the microbial community present in children without CF. We have demonstrated a degree of dissimilarity between the CF airway and control airway and have isolated key differences that suggest early signs of disruption in the homeostasis of the CF airway microbial flora.

Microbial diversity measured in sputum from adults with CF has been shown to be significantly reduced when compared to controls [19]. Our results corroborate these findings in BAL from clinically stable patient populations under 10 years of age, suggesting that the microbial community of the CF airway is fundamentally disrupted from an early age. Klepac-Ceraj *et al*. demonstrated an inverse correlation between sputum community complexity and age [7]. Here we did not see such correlations however this may not be evident in such a young patient cohort.

We found the Proteobacteria, Firmicutes, Bacteroidetes, an unclassified phylum and Tenericutes phyla to account for the majority of HybScores in the lower airways of children with CF. The major phyla identified in this study are consistent with other studies looking at sputum samples in individuals with CF [6], [7]. The same phyla were found to account for the majority of HybScores in the control airway microbial community. Three of these phyla, Firmicutes, Proteobacteria and Bacteroidetes, were previously reported to be the most abundant phyla in the healthy adult airway [1], suggesting that the same phyla may dominate the airways from childhood through to adulthood in healthy individuals, with Tenericutes possibly being more dominant in the airways in early childhood. At the genera level, an unclassified *Lachnospiraceae* genus, an unclassified *Rikenellaceaell* genus, *Pseudomonas*, *Streptococcus* and *Prevotella* were found to account for the majority of HybScores in the airways of children with CF and children without CF. Direct comparisons between different studies in this field can be challenging as studies often differ in the type of sample collected (oropharyngeal swabs [6], sputum [7], [20], BAL [21], explanted lung biopsies [22]), the age of patients recruited [6], [7], [21], the clinical status of patients at the time of sampling [14], [23]–[25], differential coverage of the 16s rRNA region, the metagenomic technique employed [6], [26], [27] and ultimately the taxonomic and analytical tools used to assign OTUs and interpret the data. All of these factors will influence the phylogenetic diversity reported.

The CF airway microbial community displayed a less rich and less even community distribution than the control airway microbial community. This suggests a level of symmetry in the control airways, not seen in the early CF airway microbial community. A high level of intrapatient variability of the CF airway microbial community has been reported in adult CF patients by Stressmann, F. A. *et al* [28]. Our young clinically stable patient cohort has revealed that this high intrapatient variability in microbial community structure is also evident very early in the life of CF patients. Taken together, this suggests that the microbial community in the airways of CF patients is unique to that patient and this unique or “personalized” airway microbial community emerges early in life.

We identified a number of bacterial taxa that were of significantly differential abundance between the control and CF lower airway. *Prevotella veroralis* is a Gram negative bacterium known to be present in the oral cavity of healthy individuals [29] and, in the present study, was found to be significantly less abundant or absent from the CF airway. *P. veroralis* has been identified previously in the airways of clinically stable CF adults [25], [30] however we found this bacteria to be absent from the airways of clinically stable children with CF. Another bacterium highlighted in this study was a *Corynebacterium* found to be in significantly higher abundance in the CF airway than in the control airway. *Corynebacterium* are Gram positive bacteria that are found in the environment and as part of the microflora of the human skin and mucous membranes, and certain members of this genus have been shown to become pathogenic under certain conditions. In particular, *Corynebacterium* species have been found to cause respiratory infections in children with CF [31]. The bacterial taxa outlined in this study may be descriptive of the primary discrepancies between the commensal microbial community of the healthy airways and that present in the CF airways.

Previous studies have shown the influence of *P. aeruginosa* on the CF airway microbiome. Klepac-ceraj and colleagues [6] noted that CF airway microbial communities dominated by *P. aeruginosa* were less diverse. Here we observed that CF patients with dissimilar *P. aeruginosa* culture scores had significantly dissimilar microbial community structures. Likewise, CF patients with dissimilar *S. pneumoniae* culture scores had dissimilar microbial community structures. These findings suggest that *P. aeruginosa* and *S. pneumoniae* may significantly influence the composition of the early CF airway microbial community. The influence of *S. pneumoniae* on the CF airway microbiome has not been reported before. The presence of other common CF bacteria such as *M. catarrhalis*, *S. aureus*, and *H. influenzae*, as detected by culture, did not influence community structure. *Pseudomonas* and *Haemophilus* were observed to have an inverse correlation across all airway samples suggesting these may be competing genera in the lungs.

Most preschool children with CF do not expectorate sputum, therefore cultures of upper airway throat swabs are routinely used to direct therapy towards lower airway infection. The suitability of upper airway oropharyngeal swabs as surrogates for lower airway BAL samples has long been in debate. In the present study we collected paired oropharyngeal swabs and BAL samples from 12 CF patients and compared the bacterial community's detected employing Phylochip analysis. We found the CF upper and lower airway microbial communities to be representative of each other. This is in agreement with a previously published study where the microbial communities from oropharyngeal swabs and BAL of a subset of children with CF were compared and found to be concordant [6]. Goddard, F. A and colleagues recently published a contradictory study where they found upper airway throat swabs and sputum samples to overestimate the diversity of the CF lower airway [22]. However, complementary analysis of the relative abundance data from the Goddard, F. A. study revealed a broadly similar microbial community in the upper and lower airway [32]. Although every attempt was made in the present study to eliminate upper airway bacterial contamination during collection of BAL samples, this cannot be ruled out as a possible reason for the similarities observed in the CF upper and lower microbial communities. The fact that the upper and lower airways are biogeographically close sites should also not be ruled out as a reason for similarities in the microbial communities that populate these sites [33]. We also observed that standard culture of upper airway samples failed to detect bacteria cultivable from several lower airway samples (data not shown). This demonstrates that culture of upper airway swabs may not accurately represent the bacteria present in the lower airway of CF patients and this remains an area of uncertainty.

The G3 Phylochip detected many more bacterial species than was detected by standard culture. This is unsurprising as culture methods are designed to select for the growth of a narrow range of well-known CF pathogens. In contrast, the G3 Phylochip is capable of detecting >50,000 bacterial taxa with high-specificity probe sets. In this analysis, responsive probes were grouped into “probe sets” empirically based on both correlations in fluorescence intensities across all biological samples and the taxonomic relatedness of the probe sequences. This is a more conservative method for annotating taxa and reduces the likelihood of diversity overestimation. We then annotated empirical OTUs according to a recently described taxonomic hierarchy [34]. Numerous methods were included in this study to limit the erroneous identification of OTUs (S1 File: Supplementary Methods). Despite this caution should be taken when interpreting results and drawing final conclusions from phylochip data as for all other metagenomic techniques.

Many of the bacterial taxa detected by the phylochip in this study have not been classified down to the species and genera levels, with many eOTUs being reported as unclassified at these levels. Because two thirds of the 16S sequences in GenBank are only classified to the Kingdom level, other 16S databases such as the Ribosomal Database Project, SILVA and Greengenes [35] (used in this study), have emerged to classify a higher proportion of 16S sequences. However many sequences still remain unclassified. Additionally, grouping of rOTUs into eOTUs based on taxonomic relatedness and fluorescence similarities across the sample set further reduces our ability to identify most eOTUs down to the species or genus level. For these reasons, taxonomic resolution using 16S rRNA-based techniques can be limiting and obtaining culture data alongside new molecular techniques, such as 16S rRNA microarrays and 454 sequencing, is of great value.

One of the key strengths of this study lies in the patient selection. The children with CF were clinically stable, unlike other studies looking at lower airway infection in CF where samples are collected during exacerbations or when individuals were clinically unwell. Considering this and the inclusion of a control group in this study, our results reveal fundamental disruptions in the early CF airway microbial community. Our study used bronchoscopy and BAL (considered the reference standard technique) to detect organisms in the lower airway of patients with CF. In addition we employed a stringent protocol designed to limit contamination from the upper airway. Few studies have investigated the microbial community of the CF lower airway and even fewer have reported on the lower airway microbiota of children without CF. Our control group consisted of children presenting with a prior history of isolated haemoptysis or stridor, without current symptoms and a subsequently discovered normal differential cell count in their BAL sample. It is a limitation of our study that our control cohort had a history of significant respiratory symptoms to the extent that they required a bronchoscopy. Despite this, we described patterns in the microbiomes that were distinct between the two cohorts. We acknowledge that the patient cohorts in this study are small however statistically significant conclusions were reached. Our data is cross-sectional, and so we cannot comment on the development of the microbial community over time. Further longitudinal studies on larger cohorts of children with CF will be of great benefit to our understanding of the CF airway microbial community and its involvement in disease progression.

This study contributes to the current knowledge of microbial communities in the airways of children with CF. We have highlighted the existence of a sizeable microbial community in the airways of control children and outlined key differences between this airway microbiome and that of the CF airway. Further studies are required to understand how changes in the CF airway microbial community impact on the airway environment and how this influences lung physiology and ultimately clinical status.

## Supporting Information

S1 File **Supplementary Methods.**
(PDF)

S2 File The 237 taxa of significantly differential abundance between the control and CF lower airways using the Welch test (p<0.05).
(XLS)

S3 File Permutations of sample group assignments for each factor in 100 FilterFactors, red is the true value. Each open circle represents a randomized permutation performed. The x-axis explains the number of randomised permutations performed and the y-axis represents the number of taxa passing the test (significantly differential abundance). Black circles represent the number of taxa found to be differential by randomized permutations and the red circle represents the number of taxa found to be differential by the original Welch test.
(PDF)

S4 File The 58 taxa present in >30% of one category and present in none of the samples from the other category.
(CSV)
