# Supplementary material for: The Microbial Community of the Cystic Fibrosis Airway Is Disrupted in Early Life
Source: PLoS One. 2014 Dec 19;9(12):e109798. doi: 10.1371/journal.pone.0109798 (PMC4272276; doi:10.1371/journal.pone.0109798)
Supplement: S1 File — Supplementary Methods. (PDF) [file pone.0109798.s001.pdf]

S1

Supplementary Methods

PhyloChip Assay Design: A full description of the PhyloChip design is provided in the supplementary methods of Hazen *et al* [1]. Briefly, sequences (*Escherichia coli* base pair positions 47 to 1473) were extracted from the NAST multiple sequence alignment available from the 16S rRNA gene database, greengenes.lbl.gov. Putative chimeric sequences were identified and removed where Bellerophon divergence ratios >=1.1 with >=90% lane-masked identity to one or both putative parents were encountered. Sequences containing three or greater homooctomers or longer or those with >=0.3% ambiguous base calls were also omitted. From the sub-alignment, putative 25-mer targets were selected with G+C of content ranging from 35-75%, secondary structure free energy (ΔG) >= -4 kcal/mol as calculated by RNAfold, complementary melting temperature of 61°C and 80°C, and self-dimerization melting temperature less than 35°C as calculated by Thermalign. Filtered rRNA gene sequences were clustered to enable selection of perfectly complementary probes representing each sequence of a cluster. Putative amplicons containing 17-mers with sequence identity to a cluster were included in that cluster. The resulting 59,959 clusters, each encapsulating an average of 0.5% sequence divergence, were considered operational taxonomic units (OTUs). The OTUs represented 2 domains, 147 phyla, 1,123 classes, and 1,219 orders demarcated within the archaea and bacteria. Each OTU was assigned to one of 1,464 families according to the placement of its member organisms in the taxonomic outline as maintained by Philip Hugenholtz [2]. The OTUs comprising each family were clustered into sub-families by transitive (single linkage) sequence identity of 72% common heptamers. Altogether, 10,993 sub-families were found.

For each OTU, multiple specific 25-mer targets were sought for prevalence in members of a given OTU but dissimilar from sequences outside the given OTU. In the first step of probe selection for a particular OTU, each of the sequences in the OTU was separated into overlapping 25-mers, the potential targets. Then each potential target was matched to as many sequences of the OTU as possible. It was not adequate to use simplistic pattern searches to match potential targets and sequences since partial gene sequences were included in the reference set. Therefore, the multiple sequence alignment provided by Greengenes was necessary to provide a discrete measurement of group size at each potential probe site. In ranking the possible targets, those having data for all members of that OTU were preferred over those found only in a fraction of the OTU members.

In the second step, a subset of the prevalent targets was selected and the probe orientation was flipped to the reverse complement to minimize hybridization to unintended amplicon. Probes presumed to be potentially problematic were 25-mers containing a central 17-mer matching sequences in more than one OTU. Thus, probes that were unique to an OTU solely due to a distinctive base in one of the four flanking bases were avoided. Also, probes with mis-hybridization potential to sequences having a common tree node near the root were favoured over those with a common node near the terminal branch. Probes complementary to target sequences that were selected for fabrication are termed perfectly matching (PM) probes. As each PM probe was chosen, it was paired with a control 25-mer (mismatching probe, MM), identical in all positions except the thirteenth base. The MM probe did not contain a central 17-mer complimentary to sequences in any OTU. The probe complementing the target PM and MM probes constitute a probe pair analyzed together. The average number of probe pairs assigned to each OTU was 37 (s.d. 9.6).

Of the features (oligonucleotides) on the array, the majority represents publicly available 16S rRNA genes, as described above. Additional probes for quality management, processing controls, image orientation, normalization controls, hierarchical taxonomic identification, or for pathogen-specific signature detection and some implement additional targeted regions of the chromosome. Probes complementary to lower confidence 16S sequences were also included to enable broadening the phylogenetic scope of analysis, when those sequences are validated with unambiguous entries into public repositories. The PhyloChip assay design includes control probes for pre-analytic, processing, pre-labelled hybridization controls, and negative controls. Pre-analytic and hybridization controls also interpretation of background signal intensity and support normalization of overall fluorescent intensity for sample to sample comparisons.

Data processing and data reductions:

Pre-processing and Data Reduction. Once OTUs are defined as present or absent as described in the main body of this manuscript, taxa are filtered to those present in at least one of the samples (Filter-1), to taxa present in samples from one category but not detected in any samples of the alternate categories (Filter-3) and to taxa significantly increased in their abundance in one category compared to the alternate categories (Filter-5). For Filter-3, the percent prevalence required among the samples in one state is first set to 100% but then iteratively decreased until the set of passing taxa intersects all samples. This ensures that each sample contains a present call for at least one of the passing OTUs. For Filter-5, the parametric Welch test was employed to calculate p-values. As this is an exploratory analysis, false discovery rates are not considered in the p-value calculations. For this study additional Benjamini-Hochberg

and permutation tests were performed to account for false discovery rates and multiple testing.

Summarization

After the taxa are identified for inclusion in the analysis, the values used for each taxa-sample intersection are populated in two distinct ways. In the first case, the Abundance metrics are used directly (AT). Note that sub-detection abundance values are not discarded. In the second case, Binary metrics are created where 1's represent presence, 0's indicate absence (BT).

Sample-to-Sample Distances Function

All profiles are inter-compared in a pair-wise fashion to determine a dissimilarity score and store it in a distance matrix. The distance functions are chosen to allow similar biological samples to produce only small dissimilarity scores. The Unifrac distance metric, as described by Lozupone et al. [3], utilizes the phylogenetic distance between OTUs to determine the dissimilarity between communities. For weighted Unifrac, the OTU abundance is additionally considered.

References

1. Hazen, T.C., et al., *Deep-sea oil plume enriches indigenous oil-degrading bacteria.* Science, 2010. **330**(6001): p. 204-8.
2. Hugenholtz, P., *Exploring prokaryotic diversity in the genomic era.* Genome Biol, 2002. **3**(2): p. REVIEWS0003.
3. Lozupone, C., M. Hamady, and R. Knight, *UniFrac--an online tool for comparing microbial community diversity in a phylogenetic context.* BMC Bioinformatics, 2006. **7**: p. 371.
