# Supplementary material for: The Microbial Community of the Cystic Fibrosis Airway Is Disrupted in Early Life
Source: PLoS One. 2014 Dec 19;9(12):e109798. doi: 10.1371/journal.pone.0109798 (PMC4272276; doi:10.1371/journal.pone.0109798)

**permutations of sample group assignments for each factor in mFilterFactors, red is the true value**

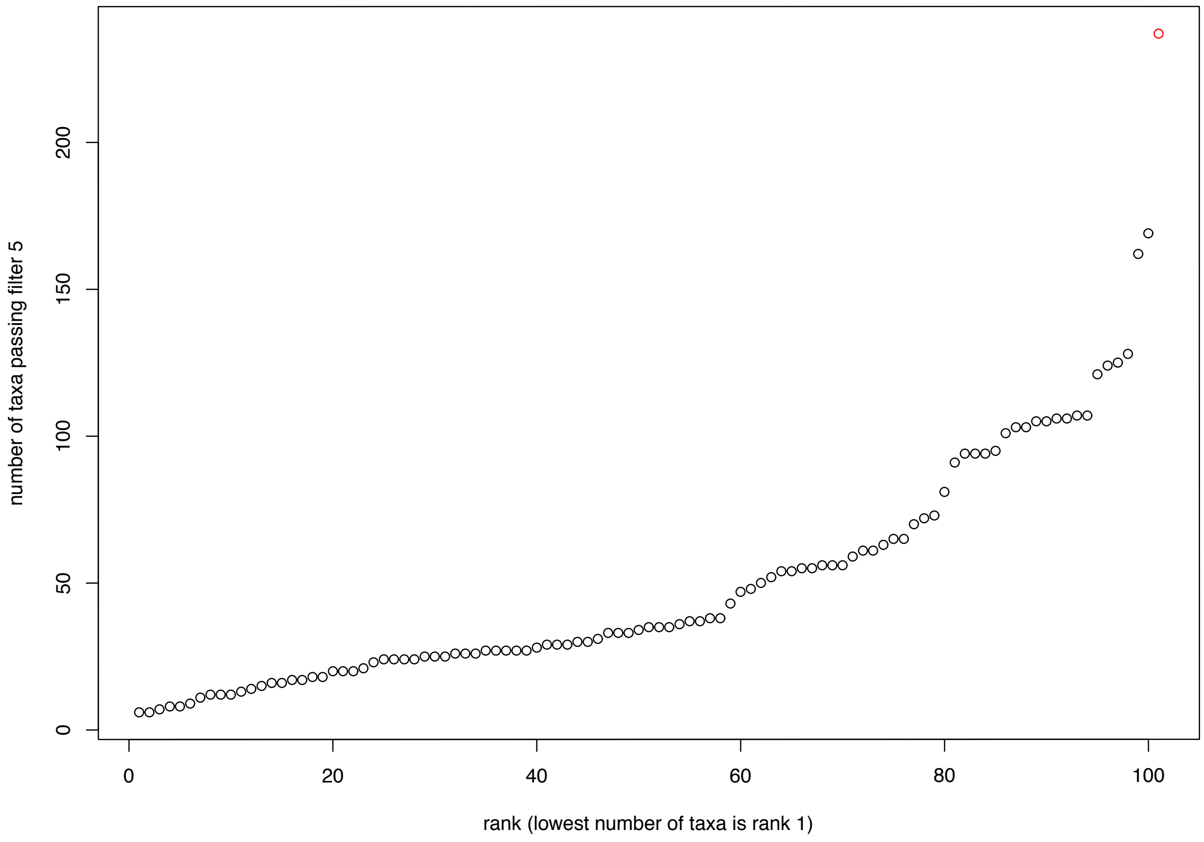

Supplement: S3 File — Permutations of sample group assignments for each factor in 100 FilterFactors, red is the true value. Each open circle represents a randomized permutation performed. The x-axis explains the number of randomised permutations performed and the y-axis represents the number of taxa passing the test (significantly differential abundance). Black circles represent the number of taxa found to be differential by randomized permutations and the red circle represents the number of taxa found to be differential by the original Welch test. (PDF) [file pone.0109798.s003.pdf]
